# GC–MS based tentative identification of γ-sitosterol from *Brassica nigra* seeds and evaluation of its anticancer potential: An integrated in vitro and in silico study

**DOI:** 10.1371/journal.pone.0351850

**Published:** 2026-06-29

**Authors:** Sajidur Rahman Akash, Tawsif Al Arian, Sumaya Alam Mim, Md. Raihan Chowdhory, Moushumi Afrin Eva, Dipalok Karmaker, Afrin Akter, Md Asadujjaman, Lamia Nur, Bijoy Kumer Ghosh, Chayan Talukder, Md. Torequl Islam, Md. Sarafat Ali

**Affiliations:** 1 Department of Pharmacy, Bangladesh University, Dhaka, Bangladesh; 2 Department of Pharmacy, Jahangirnagar University, Dhaka, Bangladesh; 3 Department of Pharmacy, University of Asia Pacific, Dhaka, Bangladesh; 4 Department of Pharmacy, University of Dhaka, Dhaka, Bangladesh; 5 Department of Medicine, Holy Family Medical College and Hospital, Dhaka, Bangladesh; 6 Pharmacy Discipline, Khulna University, Khulna, Bangladesh; 7 Department of Pharmacy, Independent University, Bangladesh; 8 Department of Biotechnology and Genetic Engineering, Gopalganj Science and Technology, University, Gopalganj, Bangladesh; 9 Department of Pharmacy, Gopalganj Science and Technology University, Gopalganj, angladesh; Banaras Hindu University, INDIA

## Abstract

Cervical cancer remains a significant global health burden, particularly in developing countries where limited access to effective therapies contributes to high morbidity and mortality. Natural products derived from medicinal plants have emerged as promising sources of novel anticancer agents; however, identifying active compounds and elucidating their molecular mechanisms remain challenging. This study investigates the anticancer potential of *Brassica nigra* seed extract, with particular emphasis on γ-sitosterol as a bioactive compound relevant to cervical cancer therapy. Isopropanol extracts of *B. nigra* seeds were analyzed using GC–MS for phytochemical profiling. Cytotoxicity was assessed via MTT assay on HeLa (cervical cancer) and Vero (normal) cell lines, followed by a brine shrimp lethality bioassay. Anti-inflammatory activity was evaluated using protein denaturation and membrane stabilization assays, and thrombolytic activity was examined through a clot lysis assay. An integrated in silico approach was employed to evaluate γ-sitosterol, including oral drug-likeness prediction, target screening, protein–protein interaction network analysis, BRCA expression profiling, molecular docking, and 100 ns molecular dynamics simulations to explore its interaction with neuropilins and other cancer-related targets. The extract demonstrated dose-dependent cytotoxicity against HeLa cells (CC₅₀ = 0.36 mg/mL), while showing lower toxicity in Vero cells (CC₅₀ = 1.26 mg/mL), yielding a Selectivity Index of approximately 3.5, indicative of selective anticancer activity. Significant inhibition of protein denaturation (IC₅₀ = 74.8 µg/mL) suggested notable anti-inflammatory potential. GC–MS identified γ-sitosterol as a major constituent (17.33% peak area). Computational analyses revealed stable binding interactions of γ-sitosterol with key targets including TP53, AKT, and BRCA1, supporting its potential role as a multi-target modulator of apoptosis, survival signaling, and genomic stability pathways. Overall, *B. nigra* seed extract, enriched with γ-sitosterol, exhibits promising cytotoxic and anti-inflammatory activities. Further isolation, mechanistic validation, and in vivo studies are warranted to confirm its therapeutic potential in cervical cancer management.

## 1. Introduction

Cervical cancer is still a major health problem around the world, especially for women in developing countries. This shows how important it is to find new ways to treat it [1,2]. The World Health Organization (WHO) and the Global Cancer Observatory (GLOBOCAN 2022) say that cervical cancer is still the fourth most common cancer in women around the world. In 2022, the disease killed an alarming 348,874 women, which is one death every two minutes. It also gave 662,301 women a new diagnosis.

Current clinical management primarily relies on chemotherapy, radiotherapy, and surgical interventions; however, these approaches are often associated with significant limitations, including systemic toxicity, non-selective damage to healthy tissues, and the emergence of multidrug resistance (MDR) [3,4]. Consequently, the development of novel therapeutic agents that are both effective and less toxic remains a critical priority in cancer research [5].

Natural products have long been recognized as an important source of bioactive compounds with diverse pharmacological properties, including anticancer activity [6]. Among these, Brassica nigra (black mustard) is known to contain a wide range of phytochemicals such as sterols, fatty acids, and phenolic compounds, which have been associated with antioxidant, anti-inflammatory, and anticancer effects [7]. However, the potential of B. nigra seed extract in cervical cancer and the underlying molecular mechanisms of its bioactive constituents remain insufficiently explored.

Phytosterols, particularly γ-sitosterol, have been reported to exhibit various biological activities, including antiproliferative, anti-inflammatory, and immunomodulatory effects in different experimental models. Nevertheless, their precise role and mechanism of action in cervical cancer are not yet fully understood. Given the complexity of cancer biology, it is increasingly recognized that effective therapeutic agents often act through multiple molecular targets rather than a single pathway.

In this context, integrative approaches combining experimental and computational techniques have emerged as powerful strategies for elucidating the multi-target mechanisms of natural compounds. Computational methods such as network pharmacology, molecular docking, and molecular dynamics (MD) simulations enable the identification of potential targets, prediction of ligand–protein interactions, and evaluation of binding stability, thereby providing mechanistic insights at the molecular level [8]. These approaches are particularly relevant for understanding complex biological processes such as apoptosis, survival signaling, and tumor microenvironment interactions in cancer progression [9–11].

Although previous studies have reported the biological activities of B. nigra and its phytochemical constituents, a comprehensive investigation integrating experimental validation with advanced computational analysis in the context of cervical cancer remains limited. Therefore, the present study aims to evaluate the anticancer potential of B. nigra seed extract and to explore the possible molecular mechanisms of its bioactive constituents using a combined in vitro and in silico approach.

## 2. Materials and methods

### 2.1. Plant collection and identification

Black mustard (*B. nigra*) seeds were sourced in August 2024 from a local market in Gopalganj, Bangladesh. The species was taxonomically verified and authenticated by the Bangladesh National Herbarium (Specimen: DACB Accession No. 135388).

### 2.2 Preparation of *B. nigra* Seed Extract

Unprocessed seeds of *B. nigra* to get rid of small particles and other debris, *B. nigra* were first cleaned with stainless steel sieves. To make a coarse powder, an electric grinder was used to mix together 1,000 grams of cleaned seeds. The powders were mixed with isopropyl alcohol at room temperature (25 ± 2°C) for 15 days, shaking them every now and then. After the extraction period, the filtrate was put through rotary evaporation (Buchi, Switzerland) under reduced pressure, which made a thick, gummy extract. As explained above, the cell pellet was then 10 ml of the identical buffer in resuspension, sonicated, and centrifuged at 8000g for 15 minutes. The crude extract was then stored at −4°C until it was needed. The extract was dissolved in 2.5% dimethyl sulfoxide (DMSO) before biological assays to make it easier to dissolve in water (for cytotoxic only). All of the solvents and reagents used were of analytical or laboratory grade and came from certified suppliers.

### 2.3 Toxicity Assessment

#### 2.3.1 Brine Shrimp Lethality Bioassay (BSLB).

The BSLB in this study was performed according to the methodology previously outlined by Meyer et al. [12], with minor modifications. Briefly, *Artemia salina* eggs were hatched in artificial seawater under continuous aeration and illumination at room temperature for 48 hours. After hatching, active nauplii were collected from the illuminated side of the hatching chamber using a Pasteur pipette and rinsed three times with fresh artificial brine solution to remove debris and unhatched eggs. The ethanolic extract of *B. nigra* seeds was prepared in five graded concentrations 100, 50, 25, 12.5, and 6.25 µg/mL. Vincristine sulfate was used as the positive control at concentrations of 2, 1, 0.5, 0.25, and 0.125 µg/mL. Artificial brine solution without any test substance served as the negative control.

For each concentration, ten active nauplii were transferred into test tubes containing 1 mL of the test sample or standard drug, and the volume was adjusted to 10 mL with artificial brine solution. Each concentration was tested in triplicate.

The test tubes were maintained at room temperature, and mortality was recorded at 24, 48, and 72 hours. Nauplii were considered dead if they showed no movement during observation. The percentage mortality was calculated, and LC₅₀ values were determined using non-linear regression analysis in GraphPad Prism software.

#### 2.3.2 Cell Viability test.

Cell viability was determined using the CellTiter 96® Non-Radioactive Cell Proliferation Assay (Promega, USA) following the manufacturer’s instructions. HeLa and Vero cells were seeded in 96-well plates at an appropriate density and allowed to attach overnight under standard culture conditions (37 °C, 5% CO₂). The cells were then treated with various concentrations of the extract and incubated for 24 h.

After treatment, the assay reagent was added to each well and the plates were incubated according to the kit protocol. The absorbance was measured using a microplate reader at 570 nm. Cell viability was expressed as a percentage relative to the untreated control group, which was set as 100% viability.

Each experiment was performed independently three times, and each concentration was tested in duplicate wells. The CC₅₀ values were calculated using nonlinear regression analysis based on a four-parameter logistic (4PL) dose–response model in GraphPad Prism. Statistical comparisons between treated groups and the control were performed using one-way ANOVA followed by Dunnett’s multiple comparison test. A p-value < 0.05 was considered statistically significant.

### 2.4 Anti-inflammatory Activity Assessment

#### 2.4.1 Protein denaturation test (egg albumin model).

This study was conducted in accordance with the methodology established by Dey et al. [13], with minor modifications. The reaction mixture consisted of 0.2 mL of egg albumin obtained from fresh hen’s eggs, 2.8 mL of phosphate-buffered saline (PBS, pH 6.4), and 2 mL of the test sample or reference drug at various concentrations. We used double-distilled water (DDW) as the control in the same amount. Incubation at 37 ± 2 °C for 15 minutes, then heated to 70 °C for 5 minutes. A colorimeter (LT-114, India) was used to measure the optical density (OD) at 660 nm after the sample had cooled. The vehicle was used as the blank. We used the following equation to figure out the percentage of membrane stabilization (i.e., stopping protein denaturation):


$$\begin{array}{c} \%\text{ Membrane stabilization }=\;\{({\text{OD}}_{\text{control}}-\;{\text{OD}}_{\text{test samples}})/{\text{OD}}_{\text{control}}\}\;\times \text{ 100} \end{array}$$

The half-maximal inhibitory concentration (IC_50_) values for BM, ASA, and their combinations were calculated using non-linear regression analysis in GraphPad Prism.

#### 2.4.2 Membrane Stabilization Test (HRBC Model).

This study was conducted using the model developed by Shinde et al. [14] with certain modifications. The Department of Pharmacy at Gopalganj Science and Technology University, Gopalganj (#bsmrstu-18PHR069–01), approved this study. First, 5 mL of fresh blood from a healthy human donor was mixed with dipotassium salt of EDTA (2.2 mg/mL). Then, the blood cells were spun at 3000 × g for 10 minutes to collect them. They were then washed three times with an isotonic solution (154 mM NaCl) in 10 mM sodium phosphate buffer (pH 7.4). Similarly, the cell suspension was centrifuged at 3000 × g for 10 minutes, and then it was put back into an equal amount of isotonic buffer solution. Further, 0.5 mL of the cell suspension was added to a mixture of 5 mL of hypotonic solution (50 mM NaCl) and 100 µL of the test or standard solution (6.25, 12.5, 25, 50, and 100 µg/mL) in 10 mM sodium phosphate-buffered saline (pH 7.4), as specified. The control tube had 0.5 mL of cell suspension, 5 mL of hypotonic solution, and 100 µL of distilled water (DW) in the buffer mentioned above. The reaction mixture was kept at room temperature for 10 minutes and then spun at 3000 × g for 10 minutes. Lastly, a colorimeter (AE-11M, Japan) was used to measure the supernatant’s optical density (OD) at 540 nm. The following equation was used to figure out the percentage of hemolysis that was stopped:


$$\begin{array}{c} \%\text{ Inhibition of hemolysis }=\;\{({\text{OD}}_{\text{control}}-\;{\text{OD}}_{\text{test samples}})/{\text{OD}}_{\text{control}}\}\;\times \text{ 100} \end{array}$$

The half-maximal inhibitory concentration (IC_50_) was measured utilizing non-linear regression analysis with the aid of Graph Pad Prism software.

### 2.5 Clot lysis test

This study was carried out following the previously described method by Hossain et al. [15] having minor modifications. Blood samples were obtained from three healthy human volunteers devoid of any history of hematological disorders, not utilizing oral contraceptives, and not receiving anticoagulant therapy. The study protocol was approved by the Human Ethics Committee of Gopalganj Science and Technology University (Approval No: 2025/GSTU/H-IEC/013). The blood was collected in sterilized tubes (1.5 mL each) and incubated for 45 minutes at 37 °C to allow the clot to form. After the blood had clotted, the serum was carefully removed from each tube without breaking the clot. The weight of each tube with the clot was then measured. We added different amounts of BM (6.25, 12.5, 25, and 50 µg/mL) to the labeled tubes. In terms of controls, 100 µL of streptokinase (SK) was utilized as a positive control, while distilled water (DW) functioned as a negative non-thrombolytic control. Next, all the tubes were incubated at 37°C and observed for clot lysis. After incubation, the liquid was carefully removed, and the tubes were weighed again to precise the alter in weight before and after the clot had broken down. This difference was figured out as the percentage of clot lysis.

### 2.6 Gas Chromatography-Mass Spectrometry (GC-MS) analysis

Gas chromatography-mass spectrometry (GC-MS) was used to analyze γ-sitosterol. We used an Agilent 7890A gas chromatograph system (Agilent Technologies, USA) with a 5675C Inert MSD detector that worked in Triple-Axis mode. An HP-5 MS capillary column (30 m × 0.25 mm i.d., 0.25 μm film thickness) was taken as a part of the system. The carrier gas was helium, which used to flow at a steady rate of 1.0 mL/min. To increase the temperature at 290°C, the injector worked in spitless mode. The oven temperature program was set up as follows: it started at 50°C and stayed there for 2 minutes, then increased to 300°C at 7°C/min. We were able to tell which parts were which by comparing their retention indices (RI) and mass spectra with those in reference libraries (NIST98, Wiley275, and CNRS) (Benali et al.,) [16].

Compound identification was performed by comparing mass spectra and retention indices with NIST, Wiley, and CNRS libraries. However, as GC–MS analysis was conducted without high-resolution mass spectrometry (HRMS), structural confirmation based solely on spectral matching may be ambiguous, particularly for sterol-type compounds that share similar fragmentation patterns. Therefore, the identification of γ-sitosterol should be considered tentative unless confirmed using an authentic reference standard under identical chromatographic conditions.

### 2.7 In Silico observation

#### 2.7.1 Pharmacokinetic properties and toxicity prediction.

The chemical structural formula of γ-sitosterol was obtained from PubChem (https://pubchem.ncbi.nlm.nih.gov/). Thereafter, the drug similarity and physicochemical properties were evaluated using SwissADME (http://www.swissadme.ch/), which was utilized to investigate the compounds’ ADME characteristics. Additionally, we employed Protox-II (https://tox-new.charite.de/) to evaluate the toxicity of γ-sitosterol [17].

#### 2.7.2 Prediction of γ-sitosterol targets.

SwissTargetPrediction (www.swisstargetprediction.ch) is a web-based tool that can guess what small bioactive compounds will do. It is used to find targets that are related to γ-sitosterol. To find all the possible targets of γ-sitosterol, we imported the SMILES into SwissTargetPrediction. We used the HERB database (http://herb.ac.cn/) to find the relevant targets of γ-sitosterol and make a complete list of pharmacological targets. We got these targets from the TCMID (http://www.megabionet.org/tcmid/) and TCMSP (https://tcmspw.com/tcmsp.php) databases [18]. Subsequently, the UniProt database (http://www.uniprot.org/uniprot/) was queried to obtain the gene symbols of the potential targets.

#### 2.7.3 Predict targets of γ-sitosterol against cervical cancer (CC).

We searched the following electronic databases for the term “CC”: GeneCards (https://www.genecards.org/), OMIM (https://www.omim.org/), TTD (http://db.idrblab.net/ttd/), PharmGKB (https://www.pharmgkb.org/), and DrugBank (https://go.drugbank.com/) [19]. Then, they used the UniProt database to fix the protein names and make them official names. They also found that the source of the species was human [20].

#### 2.7.4 Screening of intersection targets.

To elucidate the correlation between CC-related targets and the prospective targets of γ-sitosterol, we superimposed the CC targets with those of γ-sitosterol. We created Venn diagrams utilizing an online tool (http://bioinformatics.psb.ugent.be/webtools/venn/) [21]. The PANTHER classification system subsequently classified the γ-sitosterol-associated anti-CC targets (http://www.pantherdb.org/) a Kaplan-Meier estimate for survival analysis [22].

#### 2.7.5 Construction and analysis of PPI network.

The STRING database (https://string-db.org) was used to make the protein-protein interaction (PPI) network by entering these possible therapeutic targets of CC and specifying that the species was Homo sapiens. All interactions in each network had confidence values of 0.4 or higher, indicating medium to high confidence [23]. After that, this network was moved to Cytoscape (version 3.8.0) so that it could be seen. You can download the Cytoscape software from the Cytoscape website (https://cytoscape.org/). Nodes with higher degree values are potential targets of γ-sitosterol in the PPI network. The total score reflects how clear and prominent a node is. The 14 best targets were selected based on how well they fit the core objectives [24].

#### 2.7.6 GO and the Kyoto Encyclopedia of Genes and Genomes (KEGG) enrichment analysis.

We used the R package clusterProfiler_28 from the Bioconductor project to perform pathway enrichment analysis of green module genes using the Gene Ontology (GO) and Kyoto Encyclopedia of Genes and Genomes (KEGG). All experiments were held to a significance level of p < 0.05 [25]. The most relevant enriched GO and KEGG terms were shown using an online tool (http://www.bioinformatics.com.cn/). We used another web app, RAWGraphs (https://app.rawgraphs.io/), to create a Sankey diagram showing how targets and pathways are related [26].

#### 2.7.7 Gene expression and immune cell infiltration in CESC.

To examine the correlation between tumor-infiltrating immune cells and candidate gene expression, the transcriptional levels of PTGS2 (prostaglandin-endoperoxide synthase 2), DSPP (dentin sialophosphoprotein), and TYR (tyrosinase) were obtained from TCGA-CESC (cervical squamous cell carcinoma and endocervical adenocarcinoma) RNA-seq datasets and normalized to log₂ TPM [27]. TIMER2.0, a deconvolution-based computational pipeline, and immune infiltration levels of B cells, macrophages, CD8 ⁺ T cells, CD4 ⁺ T cells, neutrophils, and dendritic cells were used for measurement. Pearson and partial correlation methods were used to do correlation analysis. Tumor purity was taken into account to avoid bias from stromal or non-tumor contributions. We used p-values to determine what was important, and p < 0.05 was considered biologically relevant [28].

#### 2.7.8 Survival outcomes stratified by immune infiltration in CESC and BRCA-HER2.

We conducted a Kaplan–Meier survival analysis to decisively evaluate the prognostic significance and the relevance of immune infiltration in CESC and HER2-enriched breast cancer (BRCA-HER2). The division of patients was taken place into two groups for each immune subset: those with high infiltration (the top 50%) and those with low infiltration (the bottom 50%). The cumulative survival probability was graphed against the number of months until the subsequent follow-up. We used log-rank tests to compare survival curves, and we set the level of statistical significance at p < 0.05 [29].

#### 2.7.9 Impact of Copy Number Alterations (CNAs) on immune infiltration in CESC.

To evaluate the impact of somatic copy number alterations (CNAs) on immune infiltration, TCGA-CESC samples were classified into five CNA states: deep deletion, arm-level deletion, diploid/normal, arm-level gain, and high amplification. We looked at the infiltration levels of B cells, CD8 ⁺ T cells, CD4 ⁺ T cells, macrophages, neutrophils, and dendritic cells in different CNA groups. We used boxplots to show the data and statistical tests to find differences between CNA categories [30].

#### 2.7.10 Effect of TTN mutation status on immune infiltration in CESC.

To determine the immunological impact of TTN (titin) mutations, infiltration scores were compared between wild-type (WT) and mutated TTN samples in CESC. Immune subsets assessed included B cells, CD8 ⁺ T cells, CD4 ⁺ T cells, macrophages, neutrophils, and dendritic cells. Boxplots were generated to illustrate infiltration distributions, and comparisons between WT and mutated groups were performed using Wilcoxon rank-sum tests [31].

#### 2.7.11 Molecular docking.

Schrödinger made GLIDE (Grid-Based Ligand Docking with Energetics), which is a very popular molecular docking program. We used the LigPrep program to work with γ-sitosterol (CID_457801) [32]. The process involved adding hydrogen atoms, getting rid of ionic compounds, and taking in ions at a pH level of 7 ± 2.0. The Protein Preparation module was used to make 3D models of both normal and mutant protein variants. This included filling in missing side chains, adding cap termini, making disulfide bonds, adding hydrogen molecules, and putting together bond ordering. The OPLS3e force field was used to carry out the process of minimizing energy [33].

Using the proteins’ known binding sites, a grid box was made. The van der Waals default perimeter factor scaling (1.0) and the charging threshold (0.25) in the Receptor Grid Generation module were used to figure out the size of the grid. The Glide docking was done using the ligand docking program and the GLIDE XP parameters [34]. Prior to the analysis of medicinal compounds, a scaling factor of 0.80 was used to adjust the van der Waals radius, and a score threshold of 0.15 was employed as a standard criterion.


$$\begin{array}{c} \text{Docking score }=\text{ aVDW }+\text{ bCoul }+\text{ Hbond }+\text{ Metal }+\text{ Lipo }+\text{ Buryp }+\text{ Rotb }+\text{ Site} \end{array}$$

In this context, “a” and “b” represent the coefficient standards of van der Waals (VDW) and Coulomb (Coul) energies, respectively. “VDW” stands for van der Waals energy, and “Coul” stands for Coulomb energy. The terms “Hbond” and “Metal” both refer to the protein’s ability to form hydrogen bonds. The phrase “association with metal” illustrates this property. “Lipo” is the standard abbreviation for “lipophilicity.” “Buryp” is the standard abbreviation for “the penalty for a buried polar group.” “Rotb” stands for the penalty for a rotatable bond and describes how polar interactions function at the active site [34,35].

#### 2.7.12 Molecular dynamics simulation.

Assessment of the Molecular Dynamics (MD) simulations the microscopic stability, structural adaptability, and dynamic behavior of the protein and protein–ligand complexes. GROMACS 2025.1 [36] was used for all of the simulations. We used the CHARMM [37] force field to set the protein parameters, and SwissParam [38] to generate the ligand topologies. Before the simulation, the systems went through 2500 steps of energy minimization utilizing the steepest descent algorithm to get rid of steric clashes and improve geometry. The SPC water model was used to add water to each system in a cubic box with periodic boundary conditions. To ensure the overall charge was neutral, we used gmx genion to add counterions (Na⁺ and Cl^-^). After minimization, a two-step equilibration was done. The v-rescale thermostat was used to keep the temperature at 310 K during the first 100 ps NVT equilibration. After that, a 100 ps NPT equilibration was done with the Parrinello–Rahman barostat to keep the pressure at 1 bar and get the density to be equal. During equilibration, position restraints were put on the heavy atoms of the proteins. The LINCS algorithm was used to set bond constraints, and the Particle Mesh Ewald (PME) method was used to handle long-range electrostatic interactions. After equilibration, all systems underwent two unrestrained 100 ns production molecular dynamics simulations. Trajectories were saved at the correct times so they could be used for later analyses.

2.7.12.1 Root Mean Square Deviation (RMSD)

RMSD is a metric that quantifies the distance between frames. It is determined for every profile frame. The root mean square deviation of frame x is calculated.


$$\begin{array}{c} {RMSD}_{X}=\sqrt{\frac{\text{1}}{N}\sum_{i=\text{1}}^{N}{\left(\overset{\prime }{\underset{i}{r}}\left(t_{x}\right)\right)-r_{i}\left(t_{ref}\right)}^{\text{2}}} \end{array}$$

Where N is the number of selected atoms, r_i_ (t _ref_) represents the position of atom I in the reference structures denotes the position of the same atom in frame x after optimal structural superposition onto the reference. All RMSD calculations were performed using the gmx function in GROMACS2025.1 and the resulting trajectories were visualized using XmGrace.

2.7.12.2 Root Mean Square Fluctuation (RMSF)

You can use RMSF values to figure out how flexible a local area is by looking at how the residues move during the MD simulation. The Root Mean Square Fluctuation (RMSF) is a good way to describe changes that happen along the protein chain. To find the RMSF for residue i, use:


$$\begin{array}{c} {RMSF}_{I}=\sqrt{\frac{\text{1}}{T}\sum_{I=\text{1}}^{T}{\left(r_{i}(t)-(r_{i})\right)}^{\text{2}}} \end{array}$$

Where ri (t) shows the position I at time t, and ri is the average position over time. RMSF looks at how the trajectory changes around the mean, while RMSD looks at how each frame compares to a reference structure. The peaks in the RMSF profile indicate flexible regions, such as loops and N- or C-terminal segments. The lower values show more rigid, structurally stable areas of the protein.

2.7.12.3 Radius of Gyration (Rg)

The radius of gyration (Rg), the mass-weighted root mean square distance of a group of atoms from their center of mass. During MD simulations, Rg is often used to measure how compact and stable biomolecular systems are when they fold. A stable Rg profile means that the tertiary structure is still there, while big changes mean that the structure is expanding, unfolding, or reorganizing. This study calculated Rg for each trajectory frame using GROMACS analysis tools to keep an eye on how tightly packed the protein and protein–ligand complexes were over the 100 ns simulation.

2.7.12.4 Solvent Accessible Surface Area (SASA)

The gmx SASA module in GROMACS 2025.1 was used to find the Solvent Accessible Surface Area (SASA) along the 100 ns MD trajectory. SASA shows how much solvent is on the protein surface and how the structure might change. The default probe radius was set to 0.14 nm, which is the same as the radius of a water molecule. We took SASA values at regular intervals for the whole protein-ligand complex to see how surface exposure, folding stability, and solvent interactions changed over time during the simulation.

2.7.12.5 Hydrogen bond analysis

The gmx h-bond module in GROMACS 2025.1 was used to examine how hydrogen bonds formed and how stable they were over a 100 ns simulation. We used standard geometric rules to identify hydrogen bonds: the distance between the donor and acceptor must be less than or equal to 0.35 nm, and the angle between the donor and the hydrogen and the acceptor must be greater than or equal to 120°. We counted hydrogen bonds in each trajectory frame to assess the stability of the molecules and how ligand binding affected the number and location of hydrogen bonds within the protein structure.

## 3. Results

### 3.1. Toxicity assessment

#### 3.1.1. Brine shrimp lethality bioassay results.

We evaluated the cytotoxicity of the Isopropanol leaf extract from *B. niagra* (BN) and a cytotoxic standard (CS) utilising the brine shrimp lethality assay at three time intervals: 24, 48, and 72 hours. The cytotoxic evaluation (Table 1) demonstrated that, during the 24-hour interval, BN exhibited a dose-dependent lethality, reaching a maximum mortality of 40.00 ± 8.16% at a concentration of 100 mg/mL. The subsequent values were 26.67 ± 4.71% at 25 mg/mL, 16.67 ± 4.71% at 12.5 mg/mL, and 13.33 ± 4.71% at 6.25 mg/mL. The LC₅₀ value for BN at this time exceeded 100 mg/mL, signifying minimal cytotoxicity for this exposure duration. The control group CS exhibited notable toxicity, with a death rate of 83.33 ± 5.77% at 2 mg/mL and an estimated LC₅₀ of approximately 1 mg/mL. At the lowest concentration examined (0.125 mg/mL), CS induced a death rate of 23.33 ± 5.77%, demonstrating a significant cytotoxic profile.

**Table 1:** Percentage mortality of brine shrimp by the test samples and controls.

Sample	Concentration	Time	Mortality (%)	Mean ± SD	LD₅₀ (mg/mL)
**BN**	100	24h	40	40.00 ± 8.16	>100
	50	24h	40	40.00 ± 8.16	
	25	24h	26.67	26.67 ± 4.71	
	12.5	24h	16.67	16.67 ± 4.71	
	6.25	24h	13.33	13.33 ± 4.71	
**CS**	2	24h	83.33	83.33 ± 5.77	1
	1	24h	50	50 ± 10	
	0.5	24h	36.67	36.67 ± 5.77	
	0.25	24h	30	30 ± 0	
	0.125	24h	23.33	23.33 ± 5.77	
**BN**	100	48h	63.33	63.33 ± 5.77	50
	50	48h	50	50 ± 10	
	25	48h	33.33	33.33 ± 5.77	
	12.5	48h	26.67	26.67 ± 5.77	
	6.25	48h	20	20 ± 0	
**CS**	2	48h	100	100 ± 0	~0.19
	1	48h	96.67	96.67 ± 5.77	
	0.5	48h	76.67	76.67 ± 5.77	
	0.25	48h	66.67	66.67 ± 5.77	
	0.125	48h	43.33	43.33 ± 5.77	
**BN**	100	72h	96.67	96.67 ± 5.77	25
	50	72h	66.67	66.67 ± 5.77	
	25	72h	50	50 ± 10	
	12.5	72h	40	40 ± 10	
	6.25	72h	40	40 ± 10	
**CS**	2	72h	100	100 ± 0	<0.125
	1	72h	100	100 ± 0	
	0.5	72h	100	100 ± 0	
	0.25	72h	96.67	96.67 ± 5.77	
	0.125	72h	76.67	76.67 ± 5.77	

At the 48-hour interval, BN’s lethality escalated, exhibiting a mortality rate of 63.33 ± 5.77% at a concentration of 100 mg/mL, while the LC₅₀ decreased to 50 mg/mL. Mortality diminished with reduced concentrations, however remained evident even at the minimal dosage. Conversely, CS exhibited a mortality rate of 100 ± 0% at 2 mg/mL, 96.67 ± 5.77% at 1 mg/mL, and 43.33 ± 5.77% at 0.125 mg/mL, with its LC₅₀ lowered to around 0.19 mg/mL. Following 72 hours, BN exhibited a notable enhancement in cytotoxic activity, with mortality reaching 96.67 ± 5.77% at 100 mg/mL and 66.67 ± 5.77% at 50 mg/mL, resulting in an LC₅₀ of 25 mg/mL. Simultaneously, CS sustained its elevated lethality, with 100% mortality at concentrations as minimal as 0.5 mg/mL.

#### 3.1.2 MTT assay.

**Fig 1:**
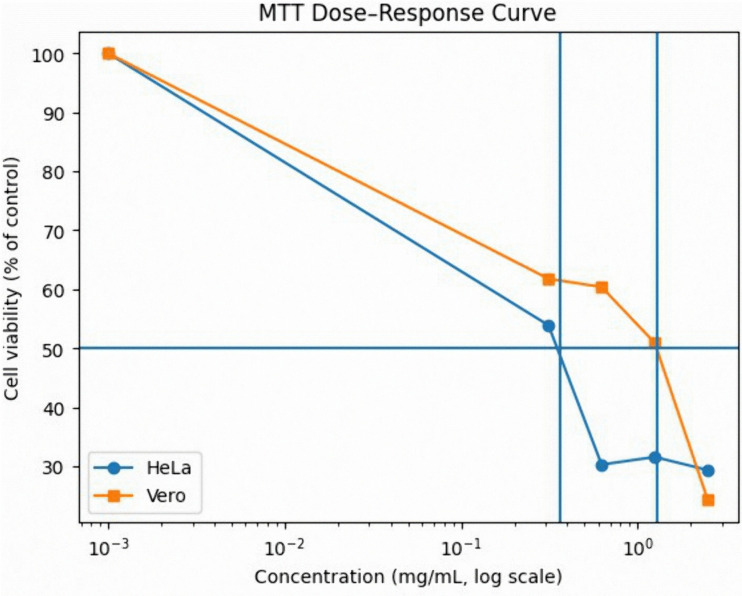
Cytotoxic effects of Brassica nigra extract on HeLa and Vero cells.

The cytotoxic activity of the extract was evaluated in HeLa and Vero cell lines using the MTT assay. Cell viability was expressed as a percentage relative to the untreated control, which was set as 100%. The extract produced a concentration-dependent reduction in cell viability in both cell lines (Fig 1).

Cell viability is expressed as a percentage relative to the untreated control (100%). Data represent mean ± SD of three independent experiments (n = 3). The calculated CC₅₀ values were 0.36 mg/mL for HeLa cells and 1.29 mg/mL for Vero cells, indicating higher sensitivity of HeLa cells to the extract. CC₅₀ values were obtained using nonlinear regression analysis based on a four-parameter logistic (4PL) model. Statistical comparisons versus control were performed using one-way ANOVA followed by Dunnett’s post hoc test (*p < 0.05).

In HeLa cells, treatment with 0.3125 mg/mL reduced cell viability to 53.89%, which further decreased to 30.27%, 31.56%, and 29.34% at concentrations of 0.625, 1.25, and 2.5 mg/mL, respectively. In Vero cells, the corresponding viabilities were 61.78%, 60.39%, 50.92%, and 24.28% at the same concentrations. CC₅₀ values were determined using nonlinear regression analysis based on a four-parameter logistic (4PL) dose–response model. The calculated CC₅₀ values were 0.36 mg/mL for HeLa cells and 1.26 mg/mL for Vero cells, indicating that HeLa cancer cells were more sensitive to the extract compared to normal Vero cells.

To further evaluate the selectivity of the extract toward cancer cells, the Selectivity Index (SI) was calculated using the formula:


$$\begin{array}{c} \text{SI }=\;{\text{CC}}_{\text{5}\text{0}}\;(\text{normal cells})\;/\;{\text{CC}}_{\text{5}\text{0}}\;(\text{cancer cells}) \end{array}$$

The calculated SI value was approximately 3.5. According to commonly used criteria, compounds with SI ≥ 3 are considered selectively cytotoxic toward cancer cells. Therefore, the obtained SI value indicates that the extract exhibits selective cytotoxicity toward HeLa cervical cancer cells compared with the normal Vero cell line.

**Fig 2:**
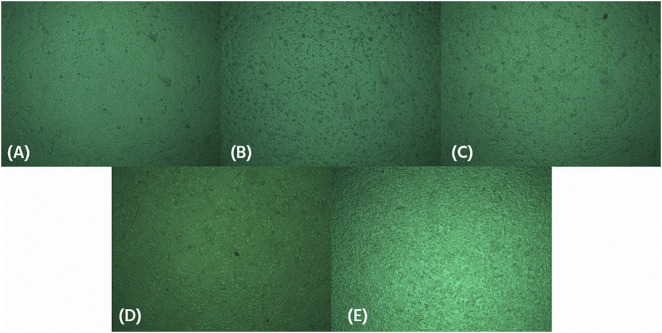
Morphological alterations in Vero cells following treatment with *B. nigra.* extract. Representative phase-contrast microscopic images showing Vero cells treated with (A) control, (B) 0.3125 mg/mL, (C) 0.625 mg/mL, (D) 1.25 mg/mL, and (E) 2.5 mg/mL extract.

In Vero cells (Fig 2), untreated control cells (A) maintained their typical elongated and adherent morphology with high confluency. Upon exposure to 0.3125 mg/mL (B), slight cellular shrinkage and partial detachment from neighboring cells were observed. At 0.625 mg/mL (C), the cells exhibited noticeable rounding, reduced confluency, and early signs of cytoplasmic condensation. Increasing the concentration to 1.25 mg/mL (D) resulted in pronounced morphological alterations, including spherical cell shapes, extensive detachment, and the presence of floating cells in the culture medium. At the highest concentration, 2.5 mg/mL (E), severe cytotoxic effects were evident, characterized by extensive cell death and fragmented cellular debris.

**Fig 3:**
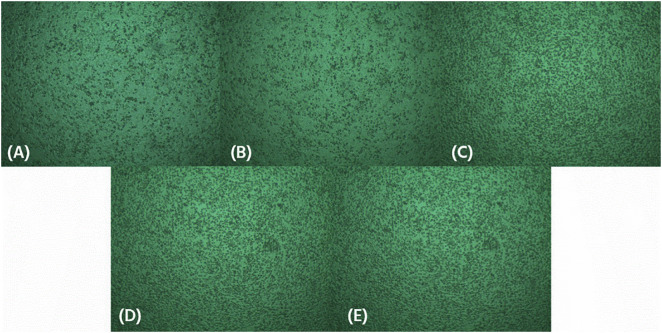
Morphological alterations in HeLa cells following treatment with *B. nigra.* Representative phase-contrast microscopic images showing HeLa cells treated with (A) control, (B) 0.3125 mg/mL, (C) 0.625 mg/mL, (D) 1.25 mg/mL, and (E) 2.5 mg/mL. Images captured under phase-contrast microscopy.

In HeLa cells (Fig 3), untreated controls (A) preserved their distinctive polygonal epithelial shape and exhibited great confluency. At a concentration of 0.3125 mg/mL (B), certain cells commenced retraction from adjacent cells and exhibited a loss of their polygonal morphology. At a concentration of 0.625 mg/mL (C), confluency significantly decreased, exhibiting nuclear condensation and the presence of floating cells. At a density of 1.25 mg/mL (D), most cells exhibited a spherical morphology, were detached, and displayed condensed cytoplasm. At 2.5 mg/mL (E), the culture exhibited significant cell mortality, with only a few fragmented cells remaining adhered. The morphological observations align with the MTT assay results, confirming that the sample induces significant, dose-dependent cytotoxicity in both Vero and HeLa cells, with HeLa cells demonstrating heightened sensitivity.

### 3.2 Anti-inflammatory activity

#### 3.2.1 Protein denaturation inhibition.

The anti-inflammatory properties of the isopropanol extract from BN were evaluated by the heat-induced egg albumin denaturation assay, utilising acetylsalicylic acid (ASA) as the positive control. We quantified the percentage of protein denaturation inhibition at concentrations between 6.25 and 100 µg/mL for both the BN extract and ASA (Table 2). The BN extract demonstrated a distinct dose-dependent enhancement in its inhibitory efficacy. At a minimal dose of 6.25 µg/mL, BN attained a 9.2 ± 1.92% suppression of protein denaturation, which dramatically increased to 74.8 ± 3.90% at 100 µg/mL. Conversely, ASA exhibited greater inhibition at all concentrations, ranging from 10.4 ± 2.97% at 6.25 µg/mL to 81.8 ± 9.65% at 100 µg/mL. The results indicate that although ASA had more pronounced inhibitory effects, the BN extract also revealed significant anti-inflammatory potential, particularly at elevated dosages. This suggests that BN may serve as a significant natural resource for the development of anti-inflammatory medicines by aiding in the stabilisation of proteins against denaturation.

**Table 2:** In-vitro Anti-inflammatory Effect of BN and ASA (acetyl salisylic acid) (Egg Albumin Denaturation Test).

Sample	Concentration (µg/mL)	% Inhibition (Mean ± SD)
**BN**	6.25	9.2 ± 1.92
	12.5	17.2 ± 3.03
	25	37.4 ± 6.39
	50	61.4 ± 5.18
	100	74.8 ± 3.90
**ASA**	6.25	10.4 ± 2.97
	12.5	24.4 ± 2.79
	25	39.6 ± 3.97
	50	73.6 ± 8.47
	100	81.8 ± 9.65

#### 3.2.2 Membrane Stabilization Test (HRBC Model).

The membrane stability test (Table 3) indicates that the vehicle control (100 µL) demonstrated 0% membrane protection, as anticipated. The test extract (BN) exhibited a concentration-dependent enhancement in protection, commencing at 12.12% at 6.25 µg/mL and to a peak of 61.85% at 100 µg/mL. Linear interpolation between the 50 µg/mL (38.01%) and 100 µg/mL (61.85%) data points produced an IC₅₀ value of around 74.12 µg/mL, signifying moderate potency. The standard chemical (CAR) exhibited a distinct dose–response relationship, with protective effects varying from 8.18% at 6.25 µg/mL to 70.00% at 100 µg/mL. The IC₅₀, determined between the 25 µg/mL (28.70%) and 50 µg/mL (52.64%) concentrations, was 47.2 µg/mL, indicating superior effectiveness relative to BN. The results indicate that although both BN and CAR can stabilize membranes under the tested conditions, CAR is more effective in attaining 50% inhibition of haemolysis.

**Table 3:** Effect of BN (test extract) and CAR (standard) on membrane stability, expressed as percentage inhibition of hemolysis at different concentrations, along with calculated IC₅₀ values. Values are mean the ± standard deviation (SD) (n = 3), One-way ANOVA followed by t-student post hoc test.

Sample/ controls	Concentration	%Membrane protection	IC_50_ [CI, R^2^]
Control (Vehicle)	100 µL		0
BN	6.25 µg/mL	12.12	74.12
	12.5 µg/mL	23.70	
	25 µg/mL	30.70	
	50 µg/mL	38.01	
	100 µg/mL	61.85	
CAR	6.25 µg/mL	8.18	47.2
	12.5 µg/mL	17.91	
	25 µg/mL	28.70	
	50 µg/mL	52.64	
	100 µg/mL	70.00	

### 3.3 Clot lysis activity

The clot lysis test demonstrated moderate thrombolytic activity of BN extract (Table 4). The percentages of clot lysis escalated with concentration, exhibiting 6.40 ± 2.13% at 6.25 µg/mL and attaining 27.89 ± 4.48% at 50 µg/mL. Although these results are markedly inferior to the positive control streptokinase (30,000 I.U.), they indicate possible cardiovascular protective actions that may enhance the anticancer capabilities of the extract. This study’s findings offer a thorough assessment of *B. niagra’s* therapeutic potential in targeting neuropilins for cervical cancer treatment. Additional study through clinical trials is required to substantiate these findings.

**Table 4:** Effect of treatments on clot lysis activity. Values are mean ± SD (n = 3); *p < 0.05 when compared to the vehicle (NC) group; ANOVA followed by Tukey post hoc test, considering p < 0.05 with a confidence level of 95%; NC (100 µL): DW (vehicle); SK (100 μL (30,000 I.U.)): SK (positive control).

Treatments	Concentration	%Clot lysis
NC	–	
SK	30,000 I.U	
BN(µg/mL)	6.25	6.40 ± 2.13
	12.5	11.87 ± 4.26
	25	14.70 ± 5.15
		27.89 ± 4.48
	50	

### 3.4 GC-MS analysis of *B. niagra* extract

**Fig 4:**
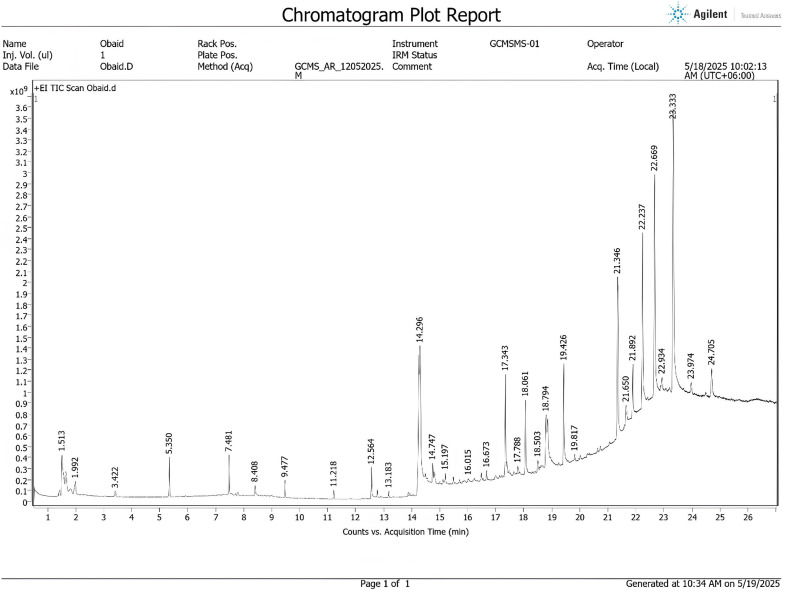
GC-MS chromatographic profile of *Brassica nigra* seed extract. Total ion chromatogram showing the detected phytochemical constituents identified by GC-MS analysis..

GC–MS analysis of the *B. nigra* seed extract tentatively identified several phytochemical constituents based on comparison of mass spectra and retention indices with reference libraries (NIST, Wiley, and CNRS) (Table 5). The major detected compounds included γ-sitosterol (17.336% area), 9,12-octadecadienoic acid (Z,Z)- (6.887% area), 6-octadecenoic acid (8.280% area), and 13-docosenoic acid methyl ester (3.142% area). Other detected constituents included eugenol (0.044% area), γ-tocopherol (6.659% area), dl-α-tocopherol (1.994% area), and (Z)-13-docosenamide (3.737% area) (Fig 4).

**Table 5:** Different types of compounds extracted in GC-MS analysis.

No.	Compound Name	RT (min)	Molecular Formula	MW (g/mol)	Peak Area (%)
1	1H-2,8a-Methanocyclopenta[a]cyclopropa[e]cyclodecen-11-one, 1a,2,5,5a,6,9,10,10a-octahydro-5,5a,6-trihydroxy-1,4-bis(hydroxymethyl)-1,7,9-trimethyl-, [1S-(1α,1aα,2α,5β,5aβ,6β,8aα,9α,10aα)]-	0.53	C20H28O6	364.19	2.34
2	10aH-2,12a-Methano-1H,4H-cyclopropa[5,6][1,3]dioxolo[2’,3’]cyclopenta[1’,2’:9,10]cyclodeca[1,2-d][1,3]dioxin-15-ol, 1a,2,7a,13,14,14a-hexahydro-1,1,6,6,9,9,11,13-octamethyl-, acetate, [1aR-(1aα,2α,7aα,7bR*,10aα,12aα,13α,14aα,15S*)]-	1.39	C28H40O6	472.28	0.03
3	(6E,10Z,14E)-6,14-Dimethyl-3-methylidene-2-oxo-3a,4,5,8,9,12,13,15a-octahydrocyclotetradeca[b]furan-10-carboxylic acid, TMS derivative	1.51	C23H34O4Si	402.22	2.99
4	Eugenol	5.93	C10H12O2	164.08	0.04
5	3-Chloro-7-hydroxy-4H-chromen-4-one, TMS	7.27	C12H13ClO3Si	268.03	0.03
6	3-Chloro-7-hydroxy-4H-chromen-4-one, TMS	7.42	C12H13ClO3Si	268.03	0.03
7	2,4-Di-tert-butylphenol	7.72	C14H22O	206.17	0.06
8	Butylated Hydroxytoluene	7.80	C15H24O	220.18	0.12
9	Phenol, 4-ethenyl-2,6-dimethoxy-	8.41	C10H12O3	180.08	0.45
10	2,2,4,6,6,8-Hexamethyl-4,8-diphenylcyclotetrasiloxane	8.89	C18H28O4Si4	420.11	0.04
11	2,2,4,6,6,8-Hexamethyl-4,8-diphenylcyclotetrasiloxane	8.99	C18H28O4Si4	420.11	0.09
12	Pentadecanoic acid, 13-methyl-, methyl ester	12.23	C17H34O2	270.26	0.02
13	9-Hexadecenoic acid, 9-octadecenyl ester, (Z,Z)-	12.37	C34H64O2	504.49	0.03
14	n-Hexadecanoic acid (n-γ-sitosterol)	12.56	C16H32O2	256.24	1.00
15	γ-Sitosterol, ethyl ester	12.89	C18H36O2	284.27	0.04
16	Isopropyl palmitate	13.18	C19H38O2	298.29	0.18
17	9,12-Octadecadienoic acid (Z,Z)-, methyl ester	13.88	C19H34O2	294.26	0.11
18	9-Octadecenoic acid, methyl ester, (E)-	13.93	C19H36O2	296.27	0.12
19	Isophytol	14.04	C20H40O	296.31	0.06
20	9,12-Octadecadienoic acid (Z,Z)-	14.26	C18H32O2	280.24	6.89
21	6-Octadecenoic acid	14.30	C18H34O2	282.26	8.28
22	Linoleic acid ethyl ester	14.48	C20H36O2	308.27	0.12
23	Isopropyl linoleate	14.75	C21H38O2	322.29	0.60
24	i-Propyl 11-octadecenoate	14.79	C21H40O2	324.30	0.32
25	9,12,15-Octadecatrienoic acid, 1-methylethyl ester, (Z,Z,Z)-	14.81	C21H36O2	320.27	0.37
26	Isopropyl stearate	15.00	C21H42O2	326.32	0.07
27	i-Propyl 7,10,13,16,19-docosapentaenoate	15.12	C25H40O2	372.30	0.18
28	cis-4,10,13,16-Docosatetraenoic acid, methyl ester	15.20	C23H38O2	346.29	0.50
29	11-Eicosenoic acid, methyl ester	15.70	C21H40O2	324.30	0.09
30	9-Hexadecenoic acid, 9-octadecenyl ester, (Z,Z)-	15.75	C34H64O2	504.49	0.02
31	2-((8Z,11Z)-Heptadeca-8,11-dien-1-yl)-4,5-dihydrooxazole	15.84	C20H35NO	305.27	0.07
32	2,3,14-Trihydroxypregn-7-ene-6,20-dione, 2TMS	16.01	C27H46O5Si2	506.29	0.19
33	8,14-Seco-3,19-epoxyandrostane-8,14-dione, 17-acetoxy-3β-methoxy-4,4-dimethyl-	16.24	C24H36O6	420.25	0.16
34	n-Propyl 11-eicosenoate	16.48	C23H44O2	352.33	0.32
35	(Z)-(Z)-icos-11-en-1-yl icos-11-enoate	16.54	C40H76O2	588.59	0.05
36	8,14-Seco-3,19-epoxyandrostane-8,14-dione, 17-acetoxy-3β-methoxy-4,4-dimethyl-	17.00	C24H36O6	420.25	0.23
37	5β-Cholestane-3α,7α,12α,24α,25-pentol TMS	17.14	C42H88O5Si5	812.55	0.11
38	(5-Acetyloxy-6-hydroxy-10a-methoxy-4,4,7,11b-tetramethyl-9-oxo-1,2,3,4a,5,6,6a,7,11,11a-decahydronaphtho[2,1-f][1]benzofuran-3-yl) acetate, TMS	17.27	C28H44O8Si	536.28	0.07
39	13-Docosenoic acid, methyl ester	17.34	C23H44O2	352.33	3.14
40	γ-Sitosterol, 2-hydroxy-1-(hydroxymethyl)ethyl ester	17.40	C19H38O4	330.28	0.29
41	Elemadienonic acid, methyl ester	17.47	C31H48O3	468.36	0.06
42	Ethyl 13-docosenoate (ethyl erucate)	17.85	C24H46O2	366.35	0.07
43	2,3-Dihydroxypropyl cis-13-docosenoate	18.06	C25H48O4	412.36	2.45
44	Glycodeoxycholic acid	18.41	C26H43NO5	449.31	0.06
45	5β-Cholestane-3α,7α,12α,24α,25-pentol TMS	18.50	C42H88O5Si5	812.55	0.45
46	Cinnamoylechinadiol, TMS	18.57	C27H40O4Si	456.27	0.03
47	1H-Naphtho[1,8a-c]furan-3(5H)-one, 7-[2-(3-furanyl)ethyl]-6,6a,7,8,9,10-hexahydro-5-hydroxy-7,8-dimethyl-, O-TMS	19.25	C23H34O4Si	402.22	0.06
48	13-Docosenamide, (Z)-	19.43	C22H43NO	337.33	3.74
49	6-[2-(3-Hydroxy-2,2,5a-trimethyl-7-methylidene-4,5,6,8,9,9a-hexahydro-3H-benzo[b]oxepin-6-yl)ethyl]-2,2,5a,7-tetramethyl-4,5,7,8,9,9a-hexahydro-3H-benzo[b]oxepine-3,4,6-triol, trimethyl ether	20.01	C33H58O6	550.42	0.13
50	Indole, 2-pentadecyl-3-phenyl-	20.63	C29H41N	403.32	0.12
51	γ-Tocopherol	21.35	C28H48O2	416.37	6.66
52	2,3-Dihydroxypropyl cis-13-docosenoate	21.65	C25H48O4	412.36	0.67
53	Retinoyl-β-glucuronide 6’,3’-lactone	22.24	C26H34O7	458.23	8.00
54	Androst-1-en-3-one, 17-(acetyloxy)-4,5-epoxy-, (4β,5β,17β)-	22.88	C21H28O4	344.20	0.04
55	Ergosta-4,7,22-trien-3-one	22.93	C28H42O	394.32	0.54
56	5’-Fluoro-2’-(trimethylsilyl)oxy-4-methoxychalcone (isomer 2) (tentative)	23.20	C19H21FO3Si	344.12	0.17
57	γ-Sitosterol (tentative)	23.33	C29H50O	414.39	17.34
58	9,19-Cyclolanost-24-en-3-ol, acetate, (3β)-	23.97	C32H52O2	468.40	0.50
59	Cholest-5-en-3-ol, 24-propylidene-, (3β)-	24.71	C30H50O	426.39	1.73
60	l-Proline, n-heptafluorobutyryl-, dodecyl ester	25.61	C21H32F7NO3	479.23	0.01

It is important to note that GC–MS-based identification was carried out using library spectral matching and retention index comparison. Since high-resolution mass spectrometry (HRMS) was not employed, compounds with similar molecular weights and fragmentation patterns may lead to ambiguous assignments. Moreover, GC–MS analysis does not reliably distinguish stereoisomers of sterol derivatives. Therefore, although γ-sitosterol showed the highest peak area (17.336%), its identification should be considered tentative. Confirmation using an authentic γ-sitosterol reference standard and co-injection under identical chromatographic conditions is required for definitive validation.

Additionally, the identification of fluorine-containing compounds (e.g., compound 58) should be interpreted with caution, as such occurrences are uncommon in plant-derived metabolites and may result from limitations of spectral library matching or analytical artifacts.

### 3.5 The pharmacokinetic properties and toxicity prediction of γ-sitosterol

The structural data for γ-sitosterol was sourced from PubChem, while the pertinent ADME information was acquired from TCMSP and swissADME. The expected pharmacokinetics of γ-sitosterol as per SwissADME, including the topological polar surface area and compliance with the Lipinski rule of five (Table 6). The findings indicated that γ-sitosterol is anticipated to demonstrate advantageous drug-likeness and comply with Lipinski’s Rule of Five. The in-silico assessment of toxicological parameters was performed utilising the ProTox-II software (Table 7). The findings indicated that γ-sitosterol did not induce any noticeable harm, with the exception of immunotoxicity.

**Table 6:** ADME analysis of γ-sitosterol from SwissADME and TCMSP database.

Name	γ-sitosterol
MW	414.71
Formula	C29H50O
Hdon	1
Hacc	1
Rbon	6
TPSA	20.23 Å²
DL	Acceptable
Lipinski	Yes (1 violation: high lipophilicity/logP)
GI absorption	Low
Caco-2 Permeability	High
BBB Permeant	No

BBB = blood brain barrier, DL = drug likeness, GI = gastrointestinal absorption, Hacc = Hydrogen bond acceptors, Hdon = hydrogen bond donors, MW = Molecular Weight, Rbon = rotatable bonds, TPSA = topological polar

**Table 7:** Computational Toxicity Risk Assessment of γ-sitosterol via ProTox-II.

LD_50_	Hepatotoxicity	Carcinogenicity	Immunogenicity	Mutagenicity	Cytotoxicity
700 mg/kg	Inactive	Inactive	Inactive	Inactive	Inactive

LD_50_ = lethal dose 50

### 3.6 Screening of targets of γ-sitosterol against CC

**Fig 5:**
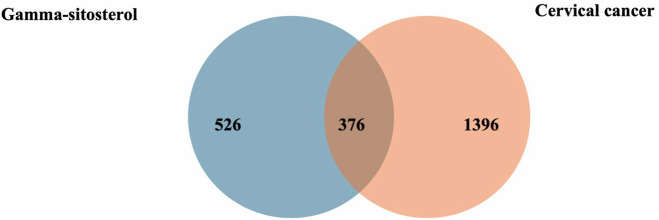
Identification of common targets between γ-sitosterol and cervical cancer.

**Fig 6:**
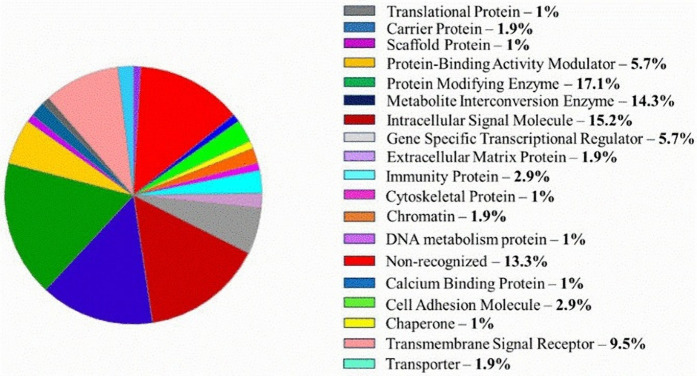
Protein class classification of intersecting target genes.

A total of 1,396 disease-associated targets were acquired from GeneCard, DrugBank, TTD, OMIM, and PharmGBK. From the aforementioned data, we discovered 12 BITC targets against CC through the intersection of 526 γ-sitosterol targets and 1396 disease-related targets (Fig 5 and Table 8). Additionally, these 12 genes were categorised into 9 unique groups utilising the Panther Classification System (Fig 6). The predominant protein classes include: cell adhesion (5.3%), defense/immunity proteins (5.3%), intercellular signalling molecules (5.3%), metabolite interconversion enzymes (42.1%), protein-modifying enzymes (21.1%), protein binding activity modulators (5.3%), scaffold/adaptor proteins (5.3%), transmembrane signal receptors (5.3%), and transporters (5.3%) (Tables 8 and 9).

**Table 8:** Profiles of hub genes and protein-coding candidates identified for potential therapeutic intervention.

Gene Symbol	Description	Category	Uniprot ID
CTSB	Cathepsin B	Protein Coding	P07858
GSK3B	Glycogen Synthase Kinase 3 Beta	Protein Coding	P49841
MME	Membrane Metalloendopeptidase	Protein Coding	P08473
PTPRC	Protein Tyrosine Phosphatase Receptor Type C	Protein Coding	P08575
CDK1	Cyclin Dependent Kinase 1	Protein Coding	P06493
BRCA1	BRCA1 DNA Repair Associated	Protein Coding	P38398
MMP3	Matrix Metallopeptidase 3	Protein Coding	P08254
MMP1	Matrix Metallopeptidase 1	Protein Coding	P03956
ELANE	Elastase, Neutrophil Expressed	Protein Coding	P08246
JAK2	Janus Kinase 2	Protein Coding	O60674
F2	Coagulation Factor II, Thrombin	Protein Coding	P00734
IRAK1	Interleukin 1 Receptor Associated Kinase 1	Protein Coding	P51617
HP	Haptoglobin	Protein Coding	P00738
PTK2	Protein Tyrosine Kinase 2	Protein Coding	Q05397
CDK4	Cyclin Dependent Kinase 4	Protein Coding	P11802
MMP2	Matrix Metallopeptidase 2	Protein Coding	P08253
CASP6	Caspase 6	Protein Coding	P55212
MMP7	Matrix Metallopeptidase 7	Protein Coding	P09237

**Table 9:** Characterization of key signaling molecules, cytokines, and growth factors involved in cellular pathways and their UniProt identifiers.

Gene Symbol	Description	Category	Uniprot ID
CCL11	C-C Motif Chemokine Ligand 11	Protein Coding	P51671
EDN1	Endothelin 1	Protein Coding	P05305
IL1A	Interleukin 1 Alpha	Protein Coding	P01583
CASP3	Caspase 3	Protein Coding	P42574
SST	Somatostatin	Protein Coding	P61278
TNF	Tumor Necrosis Factor	Protein Coding	P01375
IL18	Interleukin 18	Protein Coding	Q14116
CGA	Glycoprotein Hormones, Alpha Polypeptide	Protein Coding	P01215
BMP6	Bone Morphogenetic Protein 6	Protein Coding	P22004
LIF	LIF Interleukin 6 Family Cytokine	Protein Coding	P15018
LEP	Leptin	Protein Coding	P41159
CCL4	C-C Motif Chemokine Ligand 4	Protein Coding	P13236
AREG	Amphiregulin	Protein Coding	P15514
GHR	Ghrelin And Obestatin Prepropeptide	Protein Coding	Q9UBU3
GNRH1	Gonadotropin Releasing Hormone 1	Protein Coding	P01148
VEGFA	Vascular Endothelial Growth Factor A	Protein Coding	P15692
TGFB1	Transforming Growth Factor Beta 1	Protein Coding	P01137

### 3.7 Construction and analysis of PPI network of γ-sitosterol

**Fig 7:**
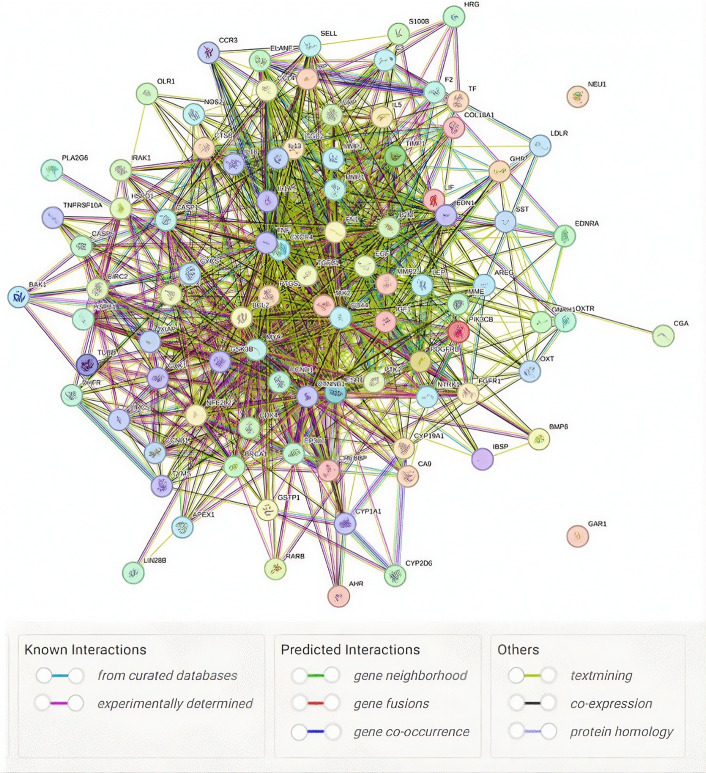
Protein–protein interaction network of common target genes.

The PPI network was established via STRING based on the acquired PPI correlations (Fig 7). The Network-Analyzer computed the degree, and the top 100 targets were identified as core targets from a total of 376 targets. The aforementioned data suggest that these main targets may be crucial in cancer therapy.

### 3.8 The GO biological process enrichment analysis

**Fig 8:**
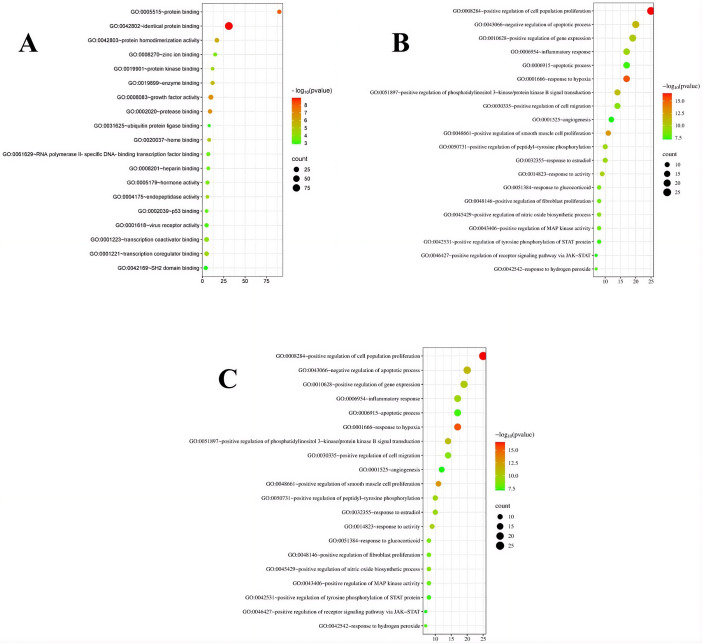
Gene Ontology enrichment analysis of common target genes.

Gene Ontology (GO) enrichment study primarily encompasses three dimensions: cellular composition, molecular function (MF), and biological process (BP). The GO enrichment entries for the biological process (BP) display the top 20. This outcome demonstrates that γ-sitosterol’s effect on CC engaged several targets and was implicated in various biological processes and molecular functions (Fig 8).

### 3.9 KEGG pathway analysis to explore the potential

**Fig 9:**
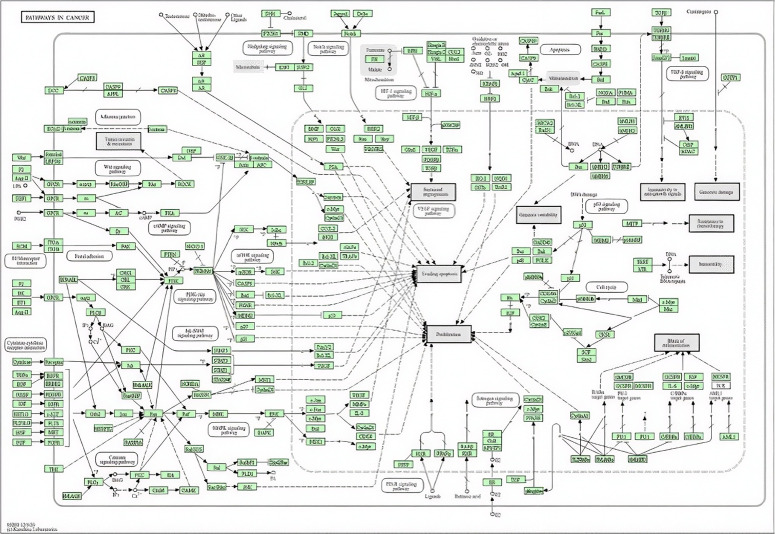
KEGG pathway enrichment analysis.

Following KEGG analysis, 120 KEGG pathways were identified, with the most enriched pathways (Fig 9). The bulk of the chosen sub-pathways pertain to three KEGG pathways: Pathway in Cancer (hsa05200), Lipid Atherosclerosis (hsa05417), and Human Papillomavirus Infection (hsa05165).

### 3.10 Bioinformatics analysis of targets of γ-sitosterol against CC

**Fig 10:**
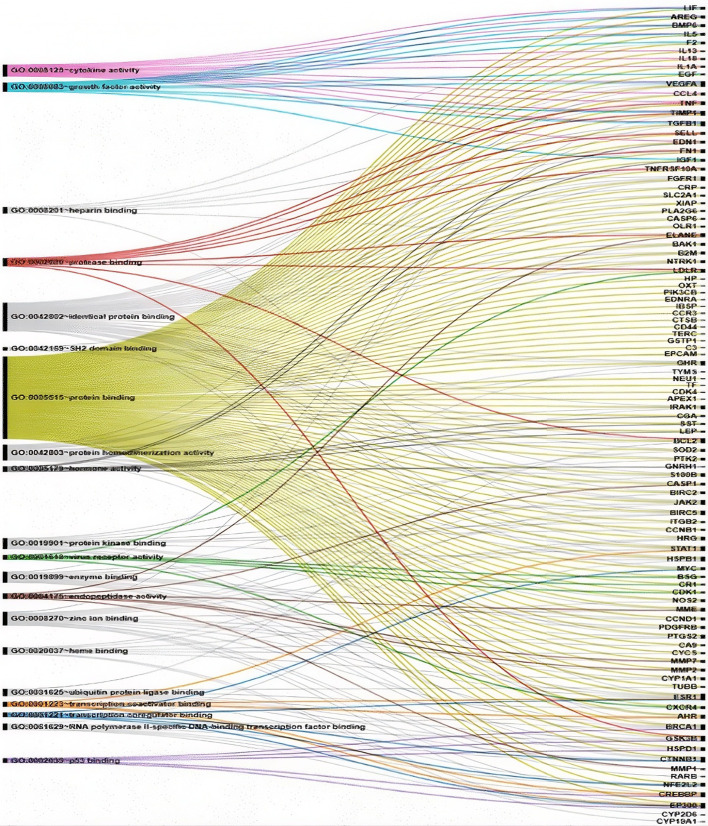
Relationship between enriched molecular functions and associated genes. Sankey diagram illustrating interactions between significant molecular functions and their corresponding target genes.

To examine the possible targets of BITC-related disease, 100 out of 376 targets shown significant correlation. Of the 376 targets, 100 were utilised to create the PPI network. The last step involved ranking these targets based on the degree score, leading to the selection of the top 20 targets associated with molecular function pathways, which included the specified gene in the Sankey diagram (Fig 10).

### 3.11 Correlation between gene expression and immune cell infiltration in CESC

**Fig 11:**
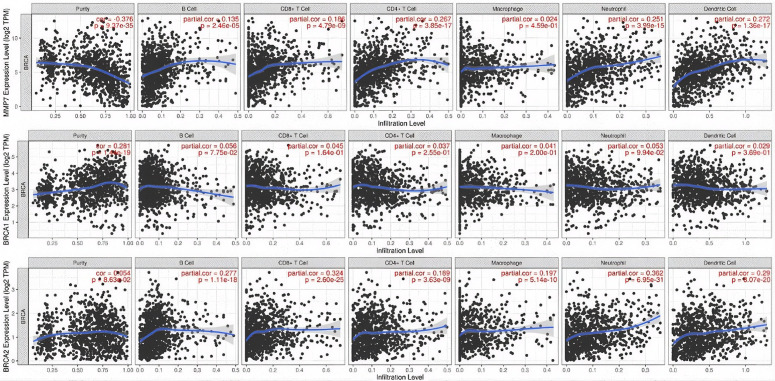
Correlation between gene expression and immune cell infiltration in BRCA.

The examination of MMP7, BRCA1, and BRCA2 expression in breast cancer demonstrated specific associations with immune cell infiltration. MMP7 exhibited a significant negative connection with tumour purity (cor = –0.376, p = 9.37e-35) and positive relationships with CD4+ T cells, neutrophils, and dendritic cells, indicating that its expression may be associated with an immunologically active tumour microenvironment. BRCA1 had a positive link with tumour purity (cor = 0.281, p = 1.49e-19) and shown very minor relationships with immune cell infiltration, indicating a restricted involvement in influencing immune responses. Conversely, BRCA2 exhibited strong positive associations with B cells, CD8+ T cells, neutrophils, and dendritic cells, signifying that BRCA2 expression is intricately linked to immune infiltration dynamics in breast cancer (Fig 11).

### 3.12 Effect of TTN mutation status on immune infiltration in BRCA

**Fig 12:**
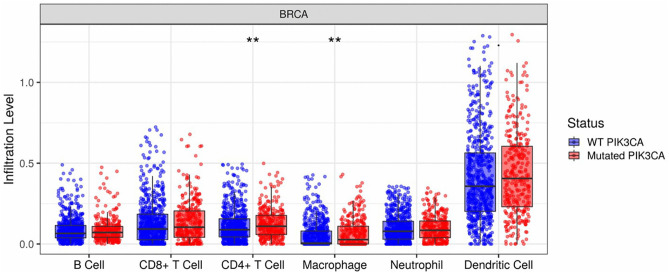
Effect of TTN mutation on immune infiltration in CESC.

Immune infiltration levels were then examined in breast cancer patients with both wild-type and mutant PIK3CA. Tumours with PIK3CA mutations demonstrated markedly increased infiltration of CD8 + T cells and CD4 + T cells relative to wild-type instances, underscoring the influence of PIK3CA mutations in enhancing T-cell mediated immune responses (p < 0.01). No significant variations were noted in the infiltration levels of B cells, macrophages, or neutrophils between the two groups, suggesting that the effects of PIK3CA mutations are restricted to T-cell subsets. Dendritic cell infiltration was continuously prevalent in both wild-type and mutant tumours, with a marginal increase observed in the mutated cohort, corroborating the notion that PIK3CA mutations augment adaptive immunological interactions within the breast cancer microenvironment (Fig 12).

### 3.13 BRCA2 expression across tumor and normal tissues

**Fig 13:**
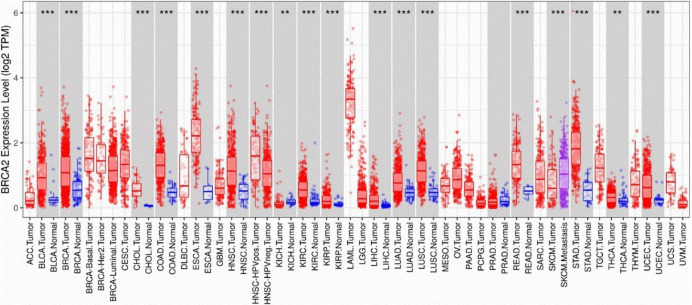
Differential expression profile of BRCA2 in cervical cancer.

The comparative expression study of BRCA2 in several tumor and normal tissues revealed a consistent pattern of overexpression in tumors. In breast cancer subgroups, such as basal-like, luminal, and HER2-positive tumors, BRCA2 expression was markedly increased relative to normal breast tissue (***p < 0.001). This tendency was not confined to breast cancer but also encompassed other malignancies, including ovarian (OV), stomach (STAD), liver (LIHC), and lung cancers (LUAD, LUSC), highlighting BRCA2’s extensive role in tumor biology. The extensive overexpression indicates that, alongside its function in DNA repair, BRCA2 may facilitate tumor growth, positioning it as a potential diagnostic and therapeutic target in several cancer types (Fig 13).

### 3.14 Molecular docking of γ-sitosterol against target proteins

Molecular docking analysis was performed using the GLIDE docking protocol to predict the binding interactions between γ-sitosterol (CID: 985) and selected cancer-associated target proteins. The docking score represents the predicted binding free energy (ΔG) of the protein–ligand complex, where more negative values indicate stronger binding affinity and greater interaction stability.

The proteins selected for this study, SNAIL1 (PDB ID: 3W5K), BRCA1 (PDB ID: 6O0K), AKT (PDB ID: 3O96), and TP53 (PDB ID: 1TUP), are known to play important roles in cancer progression. These proteins were selected because they represent key molecular regulators involved in metastasis, genomic stability, survival signaling, and tumor suppression pathways commonly dysregulated in cervical cancer. SNAIL1 is involved in epithelial–mesenchymal transition (EMT) and tumor metastasis, BRCA1 regulates DNA repair and genomic stability, AKT is a key component of the PI3K/AKT signaling pathway controlling cell survival and proliferation, and TP53 functions as a major tumor suppressor regulating apoptosis and cell cycle arrest.

The docking results demonstrated that γ-sitosterol exhibited favorable binding interactions with all selected target proteins (Table 10). The ligand showed a docking score of –5.23 kcal/mol with SNAIL1, indicating moderate interaction with this EMT-related transcription factor. A stronger interaction was observed with BRCA1 (–6.211 kcal/mol), suggesting a potential influence on DNA repair pathways. Notably, γ-sitosterol demonstrated higher binding affinity toward AKT (–7.252 kcal/mol), a key regulator of the PI3K/AKT signaling pathway. The strongest binding interaction was observed with TP53 (–7.532 kcal/mol), indicating a potentially significant interaction with this tumor suppressor protein.

**Table 10:** GLIDE docking score of protein-ligand complex.

Compound	Receptor	Binding Affinity (kcal/mol)	Grid Box	Hydrogen Bond Interaction Residue (s)	Other Interaction Residue (s)
γ-sitosterol_CID_985	SNAIL1 (RCSB ID: 3W5K)	−5.23	35.11, −14.6, −17.46	–	MET248 CYS258 ARG200
	BRCA1 (RCSB ID: 6O0K)	−6.211	−15.21, 2.26, −10.27	–	ASN143 GLY145 ARG107
	AKT (RCSB ID: 3O96)	−7.252	−8.6, −7.09, 13.15	–	TYR272 LYS268 VAL201
	TP53 (RCSB ID: 1TUP)	−7.532	−54.74, 12.17, 75.40	THR140	GLY245 LYS139 CYS176

**Fig 14:**
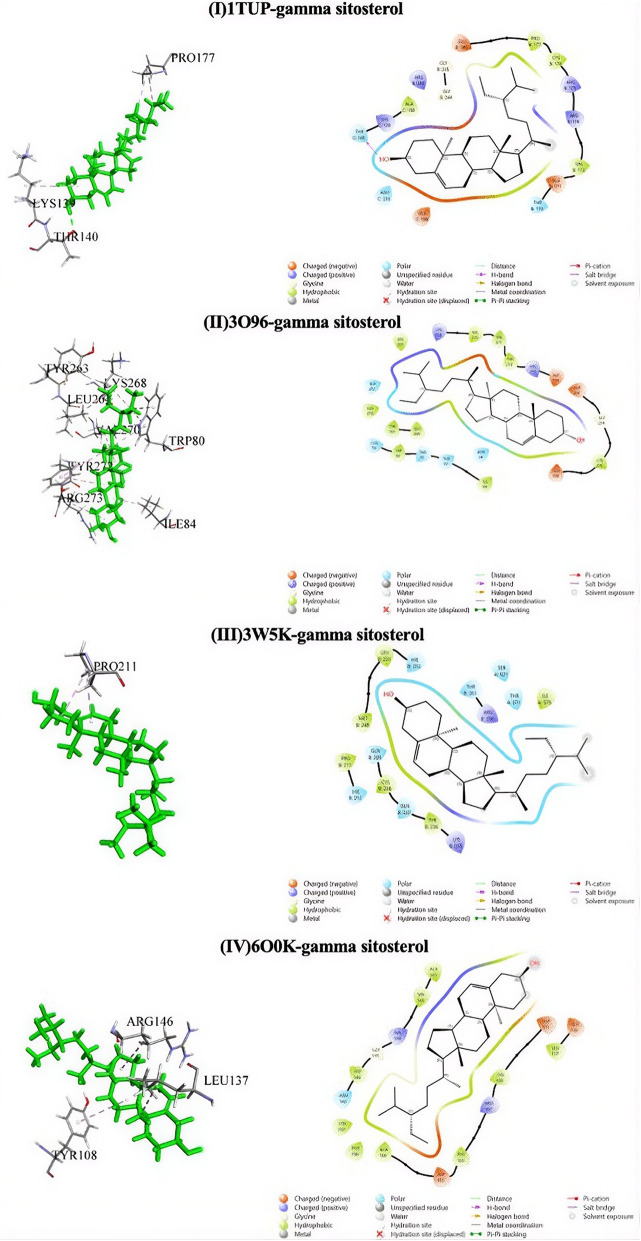
Molecular docking interactions of γ-sitosterol with selected protein targets. Three-dimensional (3D) and two-dimensional (2D) interaction maps of γ-sitosterol docked against (i) 3W5K, (ii) 6O0K, (iii) 1TUP, and (iv) 3O96.

Overall, the docking results followed the binding affinity trend:

TP53 (–7.532 kcal/mol)> AKT (–7.252 kcal/mol)> BRCA1 (–6.211 kcal/mol)> SNAIL1 (–5.23 kcal/mol), suggesting that γ-sitosterol may represent a promising candidate for further investigation with proteins involved in cell-cycle regulation, apoptosis, and cancer progression. The predicted binding interactions and docking poses of γ-sitosterol (Fig 14). To validate the docking protocol, the co-crystallized ligands of the respective proteins were redocked into their binding sites and compared with the docked pose of γ-sitosterol.

### 3.8 Molecular dynamics simulation in GROMACS

#### 3.8.1. Root Mean Square Deviation (RMSD).

**Fig 15:**
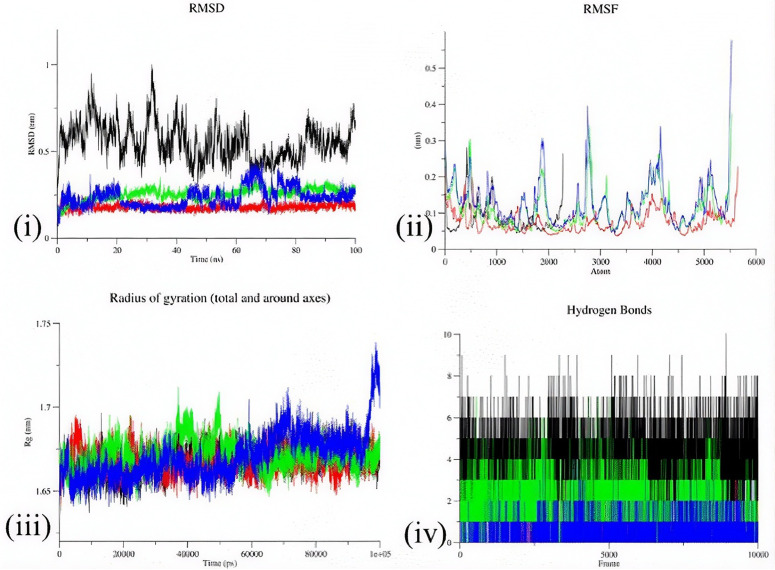
Molecular dynamics simulation analysis ofγ-sitosterol–protein complexes. Root Mean Square Deviation (RMSD), Root Mean Square Fluctuation (RMSF), Radius of Gyration (Rg), and hydrogen bond profiles for (i) 3W5K–γ-sitosterol, (ii) 6O0K–γ-sitosterol, (iii) 1TUP–γ-sitosterol, and (iv) 3O96–γ-sitosterol complexes.

Molecular dynamics simulations were performed for the same protein–ligand complexes selected in the molecular docking analysis, namely 3W5K, 6O0K, 3O96, and 1TUP. The Root Mean Square Deviation (RMSD) figure reveals that among the simulated complexes, the 3W5K–γ-sitosterol complex exhibited comparatively higher RMSD fluctuations, with values between 0.5 and 1.1 nm, indicating substantial structural alterations over time. Conversely, the remaining three structures (2QFL-γ-sitosterol, 8SKY-γ-sitosterol, and 8Q5I-γ-sitosterol) have reduced RMSD values, typically below 0.5 nm, signifying enhanced structural stability. Consequently, a plateau of RMSD values indicates that the structure fluctuates around a stable average conformation, as shown in all MD simulations (Fig 15).

#### 3.8.2. Root Mean Square Fluctuation (RMSF).

The Root Mean Square Fluctuation (RMSF) figure illustrates atomic flexibility inside the protein, with variations ranging from 0.1 to 0.5 nm. The structures of 8SKY-γ-sitosterol and 8Q5I-γ-sitosterol have marginally elevated peaks at particular atomic locations, signifying localised flexibility.

Lower RMSF values signify movements that are more constrained relative to average locations during the simulation, whereas higher RMSF values imply greater flexibility in movements (Fig 15).

#### 3.8.3. Radius of Gyration (Rg).

The Radius of Gyration (Rg) plot, which quantifies protein compactness, indicates that all structures exhibit an average Rg of around 1.65 nm, reflecting consistent compactness for the majority of the simulation (Fig 15). The 7S3L-γ-sitosterol structure exhibits an increase in Rg above 1.75 nm towards the conclusion, indicating a possible unfolding event.

#### 3.8.4. Hydrogen bonds.

Research on hydrogen bonds may promote modifications to a lead molecule to enhance its activity and provide essential insights into the stability of a ligand-protein complex. Hydrogen bonds are crucial for ligand binding (Fig 15). Given that hydrogen bond formation substantially influences drug selectivity, metabolism, and adsorption, it is essential to consider this factor. Four kinds of hydrogen bonds occur between a ligand and a protein: side-chain donor, side-chain acceptor, backbone donor, and backbone acceptor. Protein-ligand hydrogen bonds are presently characterised by the following geometric parameters: a distance of 3.5 Å between the donor and acceptor atoms (D—H···A); an angle of ≥120° for the donor-hydrogen-acceptor atoms (D—H···A); and an angle of ≥90° for the acceptor angle between the hydrogen-acceptor-bonded atoms (H···A—X).

## 4. Discussion

Cervical cancer remains one of the most prevalent malignancies among women worldwide despite advances in HPV vaccination and early screening programs [1]. Disease progression is driven by dysregulated proliferation, genomic instability, and persistent HPV infection, ultimately promoting invasion and metastasis [39]. Although chemoradiotherapy remains the standard treatment, systemic toxicity and multidrug resistance significantly limit therapeutic efficacy [4]. Consequently, there is growing interest in identifying multi-target natural compounds capable of modulating complex oncogenic signaling networks with reduced toxicity [6].

γ-sitosterol has been previously reported to exhibit antioxidant, anti-inflammatory, and anticancer activities in various experimental models [40]. However, a comprehensive investigation integrating experimental validation with network pharmacology and molecular dynamics simulations specifically in the context of cervical cancer remains limited. The present study addresses this gap by combining in vitro cytotoxic assessment with systems-level computational analyses to explore the mechanistic basis of γ-sitosterol in cervical cancer.

Our experimental findings demonstrated that the *B. nigra* seed extract exhibits selective cytotoxicity against HeLa cervical cancer cells compared to normal Vero cells, indicating preferential anticancer activity. Similar cytotoxic effects of Brassica species have been reported previously, largely attributed to their rich composition of phytochemicals such as glucosinolates, sterols, and phenolic compounds [6,40]. For example, previous studies have reported cytotoxic effects of Brassica nigra and related species in different cancer cell lines, with comparable dose-dependent responses, supporting the potential of Brassica-derived phytochemicals as anticancer agents [41]. In addition to cytotoxicity, the observed anti-inflammatory and moderate thrombolytic activities suggest that the extract may influence tumor-associated inflammation and thrombosis, both of which are recognized contributors to cancer progression [10,11].

Importantly, the biological activity of plant extracts is often not attributable to a single compound but rather to the synergistic or additive effects of multiple phytoconstituents, which may collectively enhance therapeutic efficacy and reduce toxicity [42,43]. To further elucidate the potential molecular basis underlying these experimental observations, integrated computational analyses were performed. Network pharmacology analysis revealed overlapping targets between γ-sitosterol and cervical cancer-associated genes, highlighting its potential polypharmacological nature [36,44]. Several identified targets are associated with inflammation, apoptosis resistance, extracellular matrix remodeling, and cell survival pathways, which are known to contribute to cervical cancer progression [45,46]. Notably, PTGS2 (COX-2) overexpression has been strongly linked to tumor-promoting inflammation and immune evasion in multiple cancers, including cervical cancer [40,47,48]. Similarly, aberrant activation of the PI3K/AKT signaling pathway is a hallmark of cancer progression and therapeutic resistance [45]. These findings suggest that γ-sitosterol may exert its anticancer effects by simultaneously modulating multiple oncogenic pathways, consistent with the emerging paradigm of multi-target therapeutics derived from natural products [6].

Importantly, immune infiltration analyses demonstrated correlations between gene expression and T-cell and B-cell infiltration patterns in cervical cancer datasets [10]. Several inflammation-related targets identified in the network analysis have previously been associated with immune modulation and tumor microenvironment regulation in cervical cancer [40]. These findings indicate that γ-sitosterol may exert indirect immunomodulatory effects within the tumor microenvironment; however, dedicated experimental validation is required to substantiate its role in antitumor immune regulation. Molecular docking analyses demonstrated favorable binding interactions between γ-sitosterol and key oncogenic proteins, particularly TP53 and AKT. These proteins play central roles in apoptosis regulation and survival signaling, respectively [9,45]. The strong binding affinity observed toward TP53 suggests a potential role in restoring tumor suppressor function, while interaction with AKT may contribute to inhibition of pro-survival signaling pathways. Similar binding patterns of phytosterols with cancer-related proteins have been reported in previous in silico studies, supporting their role in apoptosis induction and inhibition of tumor growth [49]. However, it is important to note that other phytochemical constituents present in the extract may also contribute to the observed biological effects, and their potential roles should not be excluded.

Furthermore, molecular dynamics simulations confirmed the structural stability of these protein–ligand complexes over time, reinforcing the likelihood of sustained biological interactions. Such stability is considered a key determinant of effective drug-target engagement in computational drug discovery [8,50].

While this study provides integrative experimental and computational insights, several important limitations must be critically acknowledged. The identification of γ-sitosterol was based on GC–MS spectral library matching without confirmation by high-resolution mass spectrometry (HRMS) or comparison with an authentic reference standard, which may limit structural certainty. In addition, the complex nature of the plant extract suggests that the observed biological activities may result from the combined or synergistic effects of multiple constituents, and the specific contribution of γ-sitosterol cannot be considered in isolation.

Furthermore, the biological validation was confined to a single cervical cancer cell line and short-term in vitro assays, thereby limiting conclusions regarding long-term efficacy, disease heterogeneity, and translational applicability. The proposed mechanistic insights were primarily derived from computational modeling and transcriptomic correlations without direct molecular or biochemical validation. Accordingly, these findings should be regarded as hypothesis-generating rather than definitive mechanistic evidence.

Although γ-sitosterol was identified as a major constituent, definitive structural confirmation remains necessary. Future studies should incorporate co-injection with an authentic standard and complementary structural elucidation techniques such as nuclear magnetic resonance (NMR) spectroscopy and liquid chromatography–high-resolution mass spectrometry (LC–HRMS). Additionally, validation across multiple cancer models and in vivo systems will be essential to establish its translational potential.

## 5. Conclusions

This study provides an integrated experimental and computational evaluation of B. nigra seed extract, identifying γ-sitosterol as a key bioactive compound with potential relevance in cervical cancer. The extract demonstrated selective cytotoxicity against HeLa cells with lower toxicity in normal Vero cells (selectivity index ≈ 3.5), along with notable anti-inflammatory and moderate thrombolytic activities, indicating its potential to modulate tumor-associated inflammation and microenvironmental processes.

GC–MS analysis tentatively identified γ-sitosterol as a major constituent of the extract. Integrated in silico analyses revealed that γ-sitosterol interacts with multiple cancer-related targets, including TP53, AKT, and BRCA1, and is involved in pathways associated with apoptosis, survival signaling, and immune modulation. Molecular docking and molecular dynamics simulations further supported the stability of these interactions, suggesting a multi-target mechanism of action.

Collectively, these findings highlight γ-sitosterol as a promising multi-target candidate for cervical cancer therapy. However, the results remain preliminary due to limitations including tentative compound identification, reliance on a single cell line, and predominantly computational mechanistic insights. Future studies should focus on structural confirmation, validation across multiple models, and in vivo investigations to establish its translational potential.

## Abbreviation:

*B. niagra*: *Brassica niagra*
MD: Molecular Dynamics
SK: Streptokinase
SRA: Sajidur Rahman Akash
TAA: Tawsif Al Arian
SAM: Sumaya Alam Mim
MRC: Md. Raihan Chowdhory
MAE: Moushumi Afrin Eva
DK: Dipalok Karmaker
MAA: Most. Afrin Akter
MA: Md Asadujjaman
LA: Lamia Nur
BKG: Bijoy Kumer Ghosh
CT: Chayan Talukder
MTI: Md. Torequl Islam
MSA: Md. Sarafat Ali.
